# The SIMPLIFY Protocol: A Monophasic Extraction System Suitable for Exposomics, Metabolomics, Lipidomics, and Proteomics Research

**DOI:** 10.1021/acs.analchem.5c05322

**Published:** 2025-11-19

**Authors:** Anh Hoang Nguyen, Victor Castro-Alves, Emilia Holmström Flores, João Marcos G. Barbosa, Matilda Kråkström, Päivikki Reinivuori, Otto Kauko, Alex Dickens, Matej Orešič, Tuulia Hyötyläinen

**Affiliations:** a Man-Technology-Environment (MTM) Research Centre, School of Science and Technology, 6233 Örebro University, Örebro SE-701 82, Sweden; b Turku Centre for Biotechnology, University of Turku and Åbo Akademi University, Turku FI-20520, Finland; c Department of Chemistry, 8058 University of Turku, Finland, Turku FI-20500, Finland; d Department of Life Technologies, 8058 University of Turku, Turku FI-20014, Finland; e School of Medical Sciences, Faculty of Medicine and Health, 6233 Örebro University, Örebro SE-701 82, Sweden

## Abstract

Advancing our understanding of human health and disease requires comprehensive analytical approaches capable of capturing the complex interplay between endogenous metabolism and environmental exposures. A major challenge in clinical research is the ability to capture multidimensional data, particularly a broad range of biochemical profiles, due to limitations of biological resources, time, and budget. In this study, we introduce the SIMPLIFY Protocol, a unified monophasic extraction method that enables the simultaneous extraction of chemical exogenous products and endogenous molecules. The method was evaluated against in-house extraction techniques, including protein precipitation with methanol (MeOH) and acetonitrile (ACN), and the Folch method using various sample types, particularly certified reference materials. We demonstrate that the SIMPLIFY Protocol not only performs comparably to our in-house methods but also offers enhanced versatility for additional applications such as derivatization and proteomics. The analyte abundances and reproducibility with this method strongly correlate with those from in-house-established techniques across diverse sample types. The method encompasses a broad spectrum of compounds, effectively profiling approximately 800 identified compounds, including polar compounds (e.g., amino acids), semipolar compounds (e.g., polyfluorinated compounds, bile acids, and lysophophatidylcholine), and nonpolar compounds (e.g., cholesteryl ester), with some limitations in extracting triacylglycerols. By maintaining simplified workflow and minimizing biological and resource consumption of multiple extractions, this method supports high-throughput exposomics/metabolomics and lipidomics studies. Furthermore, its streamlined design facilitates (semi)­automation, making it highly suitable for large-scale clinical studies, where efficiency, cost-effectiveness, and sample availability are critical factors.

## Graphical abstract

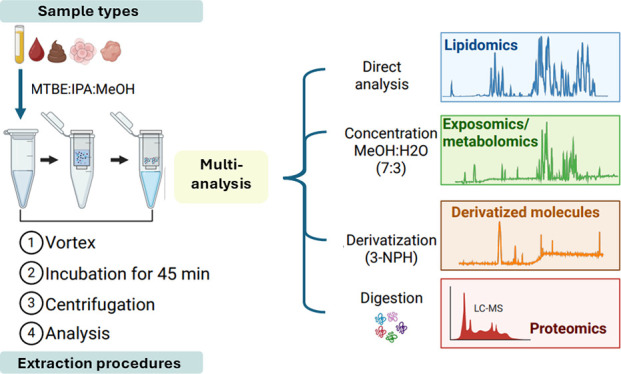

## Introduction

Exposome research offers insights into how exposures to chemical compounds shape human metabolism and mediate disease risks,^1^ underscoring the need for approaches capable of detecting exogenous compounds alongside endogenous metabolites including lipids and other small molecules.^2^

Liquid chromatography (LC) coupled with high-resolution mass spectrometry (HRMS) has emerged as the main platform for exposomics studies due to a good balance of flexibility, sensitivity, and chemical coverage compared to gas chromatography (GC)-HRMS and other traditional analytical approaches. However, the analytical challenges posed by the broad chemical diversity of exogenous and endogenous compounds remain substantial.^3^ This limitation arises mainly from the dependency of separation efficiency on the LC stationary phase, which is typically optimized for either polar or nonpolar compounds. Consequently, distinct extraction systems tailored to specific chemical polarities are often required. Analyzing multiple extracts with different polarities improves chemical coverage but is time-consuming and may require large sample volumes when multiple extraction protocols are used compared with single-extraction methods. Thus, developing extraction techniques suitable for both polar and nonpolar compounds would enhance throughput and efficiency in exposomics/metabolomics and lipidomics research.

Blood-derived samples are widely used because they reliably reflect biological function and systemic metabolism while being less invasive compared with tissue samples.^4^ Because the volume of biological samples available for research is often limited—particularly for biofluids stored in biobanks—it is essential to maximize the range of compounds extracted from a given sample. This limitation is especially pronounced in studies involving small children, where only very small amounts of blood (e.g., from fingertip sampling) can typically be obtained. Furthermore, simple extraction procedures are desirable for clinical applications to enable seamless integration into high-throughput workflows and minimize bias from multiple preparation steps.

Many methods have been developed and evaluated for the extraction of metabolites (including lipids) based on sample type and extraction objectives.^5^ Due to the complexity and the diverse physicochemical properties, current extraction methods are often fragmented, focusing on extracting specific chemical (sub)­classes, such as polar metabolites, structural lipids (bile acids, sterols, phospholipids), and exogenous compounds.^6^ For instance, aqueous methanol (MeOH) or acetonitrile (ACN) protein precipitation is often preferred to capture a broad range of polar and semipolar metabolites such as amino acids and organic acids,^7,8^ while established protocols, including Folch, Bligh, and Dyer, and methyl-*tert*-butyl ether (MTBE) extraction are widely used for extraction of lipids.^9^ Consequently, comprehensive analysis typically requires multiple sample aliquots, which are resource-intensive and time-consuming. Additionally, two- or three-phase extraction methods are often applied to maximize compound recovery. However, while effective, they carry a risk of cross-contamination between phases, which may complicate their integration into semiautomated or fully automated workflows. In contrast, monophasic extraction offers greater compatibility with automation, an increasingly important requirement in large-scale exposomics/metabolomics and lipidomics studies.

Efforts have been made to improve the efficiency and suitability of extraction methods for clinical applications.^10–12^ Recently, a study highlighted efficiency for polar and nonpolar lipids in human platelets using a monophasic solvent mixture of MeOH/MTBE/isopropanol (IPA).^13^ Regarding the combined analysis of exogenous and endogenous metabolites, we previously developed an ACN-based sample preparation and LC-MS analysis workflow for the simultaneous analysis of per- and polyfluoroalkyl substances (PFAS), bile acids (BA), and other metabolites^14^ as well as a modified Folch for lipidomics.^15^ Building on these advancements, we adapted the MeOH/MTBE/IPA solvent ratio proposed by Fu et al.^13^ and subsequently optimized procedures to develop a modified monophasic extraction method protocol suitable for exposomics/metabolomics, lipidomics, and other purposes. We demonstrate that the optimized workflow enables simultaneous extraction of exogenous (e.g., PFAS) and endogenous molecules, spanning a broad spectrum from polar metabolites to nonpolar lipids, and proteins. The short and simple procedure facilitates semi- or fully automated workflows, enabling integrated targeted and untargeted analyses with high throughput for large-scale study designs. Thus, the proposed method was named SIMPLIFY.

## Materials and Methods

### Experimental Design

We first optimized the solvent-to-sample ratio for the monophasic solvent mixture and compared its extraction efficiency with established methods, including ACN for semipolar and polar exogenous and endogenous metabolites, MeOH for polar metabolites, and the Folch method for lipidomics. For solvent-to-sample ratio optimization, each extraction method was performed in triplicate on the same day and analyzed in the same batch by three independent researchers (*n* = 9). Extraction blanks were included in every extraction. Unless stated otherwise, a pooled human plasma quality control (QC) reference sample, collected from blood donors at Örebro University Hospital (Örebro, Sweden), was used for a comparison of extraction protocols. All extractions were prepared in triplicates.

### Reagents

Extraction solutions from established exposomics and lipidomics workflows, and the optimized/combined workflow, included the following internal standards (ISTD) for assessment of polar and semipolar compounds: tryptophan-d5, hexanoic acid-d3, 3-hydroxybutyric acid-d4, heptadecanoic acid, glycoursodeoxycholic acid (GUDCA-d4), ursodeoxycholic acid (UDCA-d4), glycocholic acid (GCA-d4), chenodeoxycholic acid (CDCA-d4), cholic acid (CA-d4), glycodehydrocholic acid (GDCA-d4), taurocholic acid (TCA-d4), glycolitocholic acid (GLCA-d4), deoxycholic acid (DCA-d4), litocholic acid (LCA d4), perfluorooctanoic acid (^13^C_4_-PFOA), perfluoroundecanoic acid (^13^C_7_-PFUndA), perfluorononanoic acid (^13^C_5_-PFNA), perfluorooctanesulfonic acid (^13^C_8_-PFOS), and perfluorohexanesulfonic acid (^13^C_3_-PFHxS). For derivatized compounds, acetic acid-d4, butyric acid-d8, propionic acid-d2, and succinic acid-d4 were used. For lipids, *N*-heptadecanoyl-*d*-erythro-sphingosylphosphoryl-choline [SM­(d18:/17:0)], *N*-heptadecanoyl-*d*-erythro-sphingosine [Cer­(d18:1/17:0)], 1,2-diheptadecanoyl-*sn*-glycero-3-phosphocholine [PC­(17:0/17:0)], 1-heptadecanoyl-2-hydroxy-glycero-3-phosphocholine [LPC(17:0)], 1-palmitoyl-d31–2-oleoyl-*sn*-glycero-3-phosphocholine [PC­(16:0/d31/18:1)], CE(17:0), TG­(17:0/17:0/17:0), and TG­(19:0/19:0/19:0) were used.

### In-House Extraction Workflow for Exposomics/Metabolomics (ExMet)

The workflow was followed as previously described.^14^ Briefly, 40 μL of sample was mixed with 480 μL of ACN containing ISTDs. After vortex mixing (30 s), the extract was kept on an ice plate (45 min), transferred to a tube fitted with a filter (0.45 μm, Costar, EPA number: 98231-UT-1), and centrifuged (3000 × *g*, 3 min). Next, a 100 μL aliquot was transferred to a vial and dried using a SpeedVac system (Eppendorf Concentrator Plus) and reconstituted with 50 μL of MeOH:H_2_O (7:3, v/v) for analysis.

### In-House Extraction Workflow for Lipidomics (LIP)

The previously described modified Folch method was selected as the benchmark workflow for lipidomics.^15,16^ Slight modifications with regard to the sample volume were made, allowing a comparison between methods. Briefly, 40 μL of the sample was mixed with 480 μL of chloroform (CHCl_3_):methanol (MeOH) (2:1, v/v) containing ISTD. After vortex mixing (30 s), the extract was maintained on an ice plate (30 min) and centrifuged (9400 × *g* for 3 min). Subsequently, 25 μL of the organic/bottom phase was transferred to a vial and diluted with 25 μL of CHCl_3_:MeOH (2:1, v/v) prior to analysis. The potential insolubility during direct analysis was evaluated, and no peak broadening or distortion at early elution times was observed, as demonstrated by the early eluting acylcarnitine peaks shown in Figure S1.

### Sample-to-Solvent Ratio Optimization of the Proposed Monophasic Extraction Workflow

Four volumes of monophasic MeOH:MTBE:IPA (20:15:15, v/v) extraction solvent (MMI), 120, 240, 360, and 480 μL, referred to as MMI_120_, MMI_240_, MMI_360_, and MMI_480_, respectively, were used to extract 40 μL of the sample. After vortex mixing (30 s), the extract was kept on an ice plate (45 min) and then transferred to a tube fitted with a 0.45 μm filter and centrifuged at 3000 × *g* for 3 min. For exposomics/metabolomics analysis, a 100 μL aliquot was transferred to a vial, evaporated in a SpeedVac system, and reconstituted with 50 μL of MeOH:H_2_O (7:3, v/v) for analysis. For lipidomics analysis, each extract was directly analyzed without additional processing.

### LC-HRMS-Based Exposomics/Metabolomics and Lipidomics

Analyses were conducted on a UHPLC 1290 Infinity II system, coupled with 6545 QTOF (Agilent Technologies, Santa Clara, California, USA) equipped with a dual electrospray ionization source. Samples were injected into an ACQUITY UPLC, BEH C18 column (2.1 × 100 mm, 1.7 μm) coupled with a BEH C18 Vanguard precolumn (Waters Corporation, Milford, Massachusetts, USA).

For exposomics/metabolomics analysis, mobile phase MP­(A) 2 mM ammonium acetate (NH_4_Ac) in H_2_O/MeOH (7:3, v/v) and (B) 2 mM NH_4_Ac in MeOH were used. The following LC gradient was applied: 0.0–1.5 min, 5% MP­(B), 1.5–4.5 min to 30% MP­(B), 4.5–7.5 min, 70% MP­(B), and 7.5–12.0 min with 100% MP­(B) until the end of the run. For lipidomics, MP­(A) was 10 mM NH_4_Ac in H_2_O containing 0.1% formic acid (FA) and MP­(B) 10 mM NH_4_Ac in ACN:IPA (1:1, v/v) containing 0.1% FA, with the following gradient: 0–2 min, 35% MP­(B), 2–7 min 80% MP­(B), 7–14 min 100% MP­(B). The flow rate (0.4 mL/min) and the column and sample manager temperatures were set at 50 and 10 °C, respectively, for both methods.

For exposomics/metabolomics, 10 μL of sample was analyzed in negative ionization mode, and for lipidomics, 1 μL was analyzed in positive ionization mode, using the following parameters: 4.5 kV capillary voltage, 1.5 kV nozzle voltage, nitrogen pressure, and sheath gas flow of 21 bar and 11 L/min, respectively, and nebulizer temperature 379 °C; acquisition rate 2 spectra/s; and mass range of *m*/*z* 70–2000. The MassHunter B.06.01 software (Agilent Technologies) was used for data acquisition.

### LC-HRMS-Based Exposomics/Metabolomics and Lipidomics Data Processing

Data were processed using MZmine 3.9 software (MZio, Bremen, Germany).^17^ The preprocessing steps included definition of noise level, construction of extracted ion chromatograms, integration of features, isotopic peak grouping, filtering, alignment, and gap filling. Detailed steps of LC-HRMS data processing are provided in Table S1 (in the Supporting Information). Metabolites and lipids were identified by using an in-house library by matching experimental *m*/*z* values and retention times (RTs) with those of reference standards. Peaks with a signal-to-noise ratio (S/N) < 3, calculated as the average peak area of all samples divided by the average peak area in blank extractions, were excluded from the data set. Peak areas were normalized to the ISTD eluting closest in RT to each compound. Among all tested methods, ISTDs exhibiting the most stable relative standard deviations (RSDs) were selected for normalization of compounds within specific retention time ranges (Table S1). Compounds with a relative standard deviation (RSD) below 30% in at least one extraction method were retained for final evaluation.

### Analysis of Certified Materials and Linearity of Matrix-Matched Calibration Curves

The most suitable sample-to-solvent ratio using the MMI extraction protocol was compared with those of ExMet and LIP using standard reference materials from the National Institute of Standards and Technology (NIST SRM; NIST, Gaithersburg, Maryland, USA). Specifically, NIST SRM 1950 and NIST SRM 1957 were used for comparison between MMI and ExMet, while NIST SRM 1950 was used for comparison between MMI and LIP with xx replicates. In addition to the analysis of certified materials, three matrix-matched calibration curves were prepared to evaluate the linearity among the extraction methods: a six-point calibration curve of five PFAS (1.5–60.0 ng/mL), 20 bile acids (20.0–640.0 ng/mL), a seven-point calibration curve of 46 polar metabolites (0.1–40.0 μg/mL) including amino acids, nucleotides, and organic acids, and a seven-point calibration curve of 14 lipids (0.1–5.0 μg/mL) representative of different lipid classes.

### Cross-Laboratory Assessment

After identifying the most suitable sample-to-solvent ratio for the optimized extraction protocol, we compared its performance with established metabolomics (MeOH) and lipidomics (same Folch method, LIP) protocols in an independent laboratory using a different instrument platform, as detailed in the Supporting Information. Briefly, for exposomics/metabolomics analysis, 40 μL of an in-house QC plasma sample was mixed with 400 μL MeOH containing IS. After vortex mixing (30 s), the extract was maintained on an ice plate (30 min), transferred to a filter tube (0.45 μm), and centrifuged (3000 × *g*, 3 min). For metabolomics analysis, a 30 μL aliquot was transferred to a vial, evaporated in a SpeedVac system, and reconstituted with 100 μL of H_2_O for analysis. For lipidomics, 10 μL of the in-house QC plasma sample was mixed with 10 μL of 0.9% NaCl 0.9% and 120 μL of CHCl_3_:MeOH (2:1, v/v) containing IS. After vortex mixing (30 s), the extract was maintained on an ice plate (30 min) and centrifuged (9400 × *g*, 3 min). Subsequently, 30 μL of the organic/bottom phase was transferred to a vial for lipidomics analysis. All extractions were prepared in triplicates. Details about the method and acquisition are available in the Supporting Information.

### Other Applications of the Proposed Monophasic Extraction Workflow (Derivatization and Proteomics)

We further evaluated the suitability of extracts obtained through the MMI_480_ extraction workflow for analysis of short-chain fatty acids (SCFAs) and tricarboxylic acid (TCA) cycle-related metabolites after derivatization with 3-nitrophenylhydrazine (3-NPH), and for proteomic analysis.

### SCFA and TCA-Related Metabolites (3-NPH Derivatization)

The MMI_480_ workflow was used to extract human whole stool research grade testing material (RGTM; NIST) from subjects eating vegan diets (RGTM 10162) and omnivore diets (RGTM 10172), which are known to contain measurable SCFAs.^18^ Three replicate samples were prepared using the extraction workflow, followed by derivatization and analysis with UHPLC-Q-TOF-MS, as described previously.^19^ Briefly, 25 μL of the extract was derivatized using a mixture of 25 μL of 50 mM 3-NPH, 25 μL of 50 mM 1-ethyl-3-(3-(dimethylamino)­propyl)­carbodiimide (EDC), and 25 μL of 7% pyridine, all in H_2_O:MeOH (3:7, v/v). After vortex mixing, the sample was incubated on an orbital shaker (450 rpm) at room temperature for 60 min. Next, 50 μL of 0.2% FA was added to stop the reaction, and samples were directly analyzed in negative ion mode.^20^ Identification was performed by matching *m*/*z* and retention time with those of derivatized (S)­LCFA (*n* = 25) and TCA-related and tryptophan metabolite (*n* = 34) analytical standards.

### Proteomics Analysis

Protein lysates from A375 melanoma cells with varying cell number (1.0 × 10^04^, 2.5 × 10^04^, and 5.0 × 10^04^ cells) were obtained using either the MMI extraction protocol or a conventional lysis with urea. Cell lysates or protein precipitates from MMI extraction were solubilized with 8 M urea in 50 mM Tris buffer at pH 8. Lysates were reduced with dithiothreitol, alkylated with iodoacetamide, and diluted to <2 M urea concentration before overnight digestion with trypsin (Promega, Madison, Wisconsin, USA) at room temperature. Following digestion, peptides were desalted with a Sep-Pak tC18 cartridge (Waters Corporation), evaporated to dryness, and stored at −20 °C. The LC-ESI-MS/MS analysis was performed on an Evosep One HPLC system (Evosep, Odense, Denmark) coupled to a timsTOF fleX MS (Bruker, Bremen, Germany) equipped with a CaptiveSpray nanoelectrospray ionization source. A detailed description of the method is provided in the Supporting Information.

### Statistical Analysis

Prior to statistical analysis, data were log-transformed (base 10) and autoscaled. Principal component analysis (PCA) was performed using MetaboAnalyst 6.0.^21^ Trend plots and scatter plots were visualized using GraphPad Prism 10. Data are presented as the mean ± standard deviation (SD) unless stated otherwise.

## Results and Discussion

A previous study suggested that a monophasic mixture of MTBE, MeOH, and IPA effectively extracts both polar and nonpolar compounds.^13^ Here, we adapted that protocol to our routine workflow and evaluated its suitability for omics applications by comparison with standard approaches for exposomics/metabolomics and lipidomics while also assessing its applicability for other applications, including proteomics. In the current study, we employed negative mode for exposomics/metabolomics and positive mode for lipidomics, which together capture the majority of compounds most relevant to our targeted coverage goals with deliberate overlap between panels.

### Optimization of the Sample-to-Solvent Ratio

We optimized the solvent-to-sample ratio (v/v) to maximize compound coverage and reproducibility using in-house plasma QC samples. The adequate solvent-to-sample ratio is particularly important for efficient extraction of nonpolar lipids, such as DGs and TGs. Four volumes of MTBE:MeOH:IPA, 120, 240, 360, and 480 μL, designated as MMI_120_, MMI_240_, MMI_360_, and MMI_480_, respectively, were tested for extracting analytes from 40 μL of plasma. The extraction efficiencies were compared with those obtained using established methods, ExMet and LIP. The optimal ratio was selected based on its ability to balance exposomics/metabolomics and lipidomics coverage while achieving results comparable to those of ExMet and LIP. This ratio was further validated by profiling certified reference materials and assessing the linearity of matrix-matched calibration curves.

### Comparison of the Results with an In-House Exposomics/Metabolomics

For exposomics/metabolomics, PCA revealed a clear discrimination among the four ratios and the ExMet method (Figure S2). Notably, samples extracted by the same methods clustered tightly together, except the MMI_120_ method, suggesting the overall robustness of metabolomics profiles across replicates. The measured intensities of ISTDs were consistent across all tested methods. At the same time, RSDs decreased markedly as the sample-to-solvent ratio increased from 1:3 to 1:12 across various chemical classes, including BA, amino acids, fatty acids, and PFAS (Figure 1A). By matching with in-house library, a total of 109 compounds were identified with RSD <30% in at least one of the five methods (Table S2). Trend plots further revealed a decreasing trend in metabolite intensity from MMI_120_ to ExMet, accompanied by an inverse trend in RSDs. Notably, MMI_480_ yielded higher metabolite intensity with RSDs comparable to those of ExMet among those ratios tested (Figure S3). This was further supported by strong correlations in peak areas between MMI_480_ and ExMet (*R* ^2^ = 0.836, Figure 1B), along with a higher number of metabolites with stable RSDs (<30%) (Figure 1C andFigure S4), supporting the suitability of MMI_480_ for exposomics/metabolomics applications. Few metabolites displayed poor correlations due to the poor detectability of the ExMet method (Table S2). Collectively, among the four sample-to-solvent ratios tested, MMI_480_ shows extraction efficiency and reproducibility comparable to those of ExMet.

**Figure 1:**
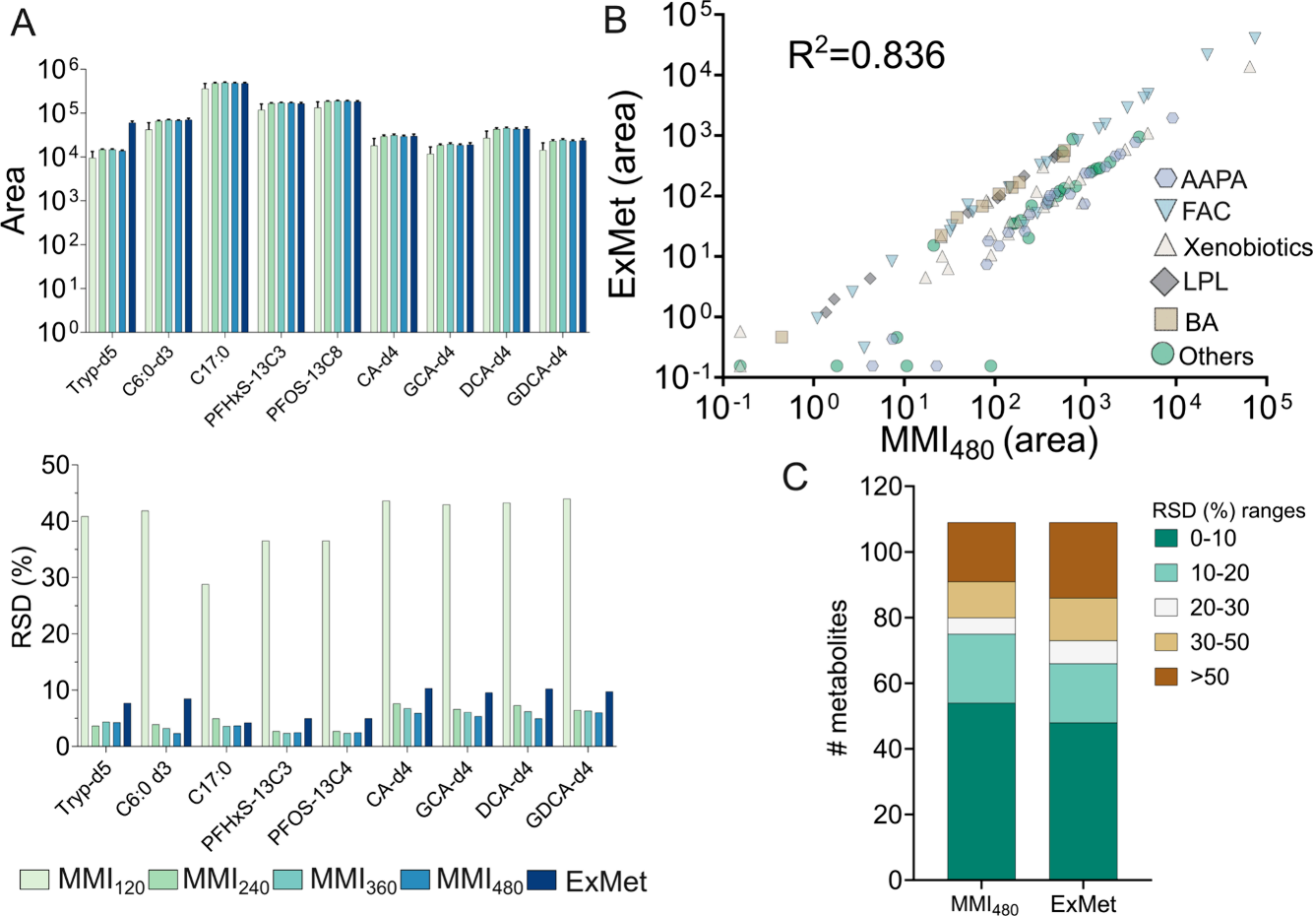
Performance of the newly developed method (MMI) compared with that of the exposomics/metabolomics method (ExMet). (A) Peak areas and relative standard deviations (RSD, %) of internal standards (ISTDs) extracted using MMI and ExMet. (B) Correlation of compound peak areas between MMI_480_ and ExMet. (C) RSD range distribution of detected compounds extracted by the two methods. Peak area was normalized and log10-transformed; zero value was imputed with half of the minimum value before log transformation. Undefined RSD values caused by nondetected compounds (zero mean value) in one of the methods were replaced with 100 as a conservative imputation. AAPA: amino acid, peptides, and analogues, FAC: fatty acids and conjugated, LPL: lysophospholipids, BA: bile acids.

### Comparison with an In-House Lipidomics

Regarding the overview of lipid profile, a clear distinct pattern was also observed across tested methods, with a higher solvent-to-sample ratio showing more tightly clustered profiles. Notably, lipid species, such as acylcarnitine, PC­(34:2­(OH)), and PE(34:0), contributed strongly to this separation (Figure S5). ISTD peak area comparisons showed that semipolar lipid ISTDs (e.g., LPC, PC) exhibited comparable peak areas across methods, whereas nonpolar lipid ISTDs (e.g., CE and TG) exhibited the highest intensity in LIP, followed by MMI_480_. Despite relatively high RSDs for certain ISTDs, such as CE(17:0), TG­(17:0/17:0/17:0), and TG­(19:0/19:0/19:0), MMI_480_ demonstrated consistent RSD values and low within-method variation (Figure 2A). Visualization of 263 identified lipids (with at least one RSD <30%) further revealed that polar lipids (e.g., LPC, PC, PE, and SM) exhibited a slightly higher peak area in the proposed extraction mixtures, with the general highest peak area and RSD values observed in MMI_120_, generally decreasing from MMI_240_ onward. In contrast, nonpolar lipids (e.g., TG and CE) were more abundant in LIP, followed by MMI_480_ (Figure S6). A closer evaluation of MMI_480_ revealed a strong correlation in lipid peak areas across lipid classes with LIP (*R* ^2^ = 0.957) and comparable RSD values (Figure 2B andFigure S7). Specifically, glycerophospholipids and sphingolipids showed improved RSDs in MMI_480_, while other lipid classes remained similar to those in LIP. In general, 90% of detected lipids had RSDs below 30% in both methods (Figure 2C), and MMI_480_ showed promising results for lipid extraction with approximately 80% of the lipids overlapping with the LIP method (Table S3). In summary, MMI_480_ demonstrated efficiency and reproducibility most similar to those of established methods (ExMet and LIP) among the tested ratios.

**Figure 2:**
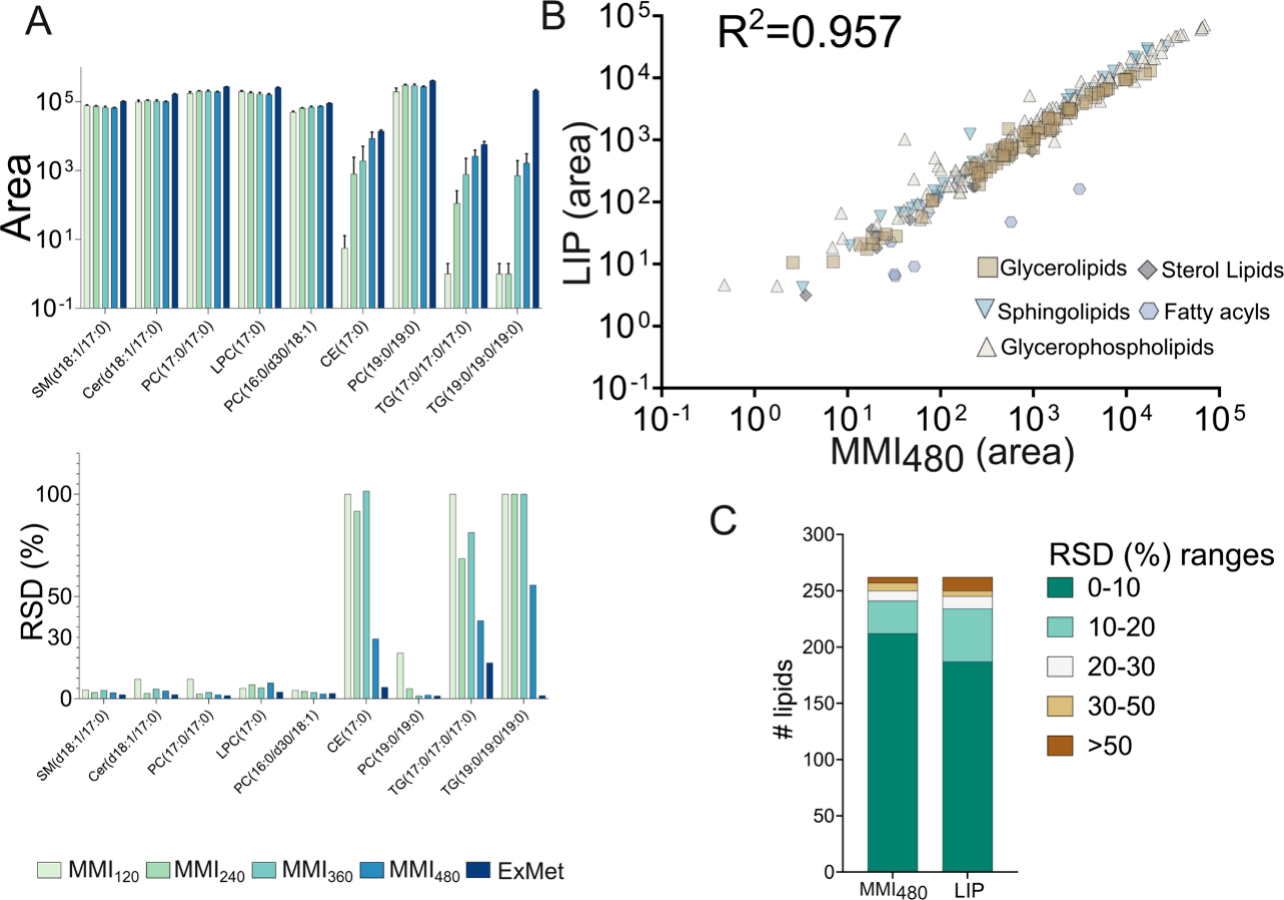
Lipidomics performance of the newly developed MMI method compared with that of the Folch method (CHCl_3_:MeOH, 2:1, v/v; LIP). (A) Peak areas and RSD (%) of internal standards (ISTDs) extracted using MMI and LIP. (B) Correlation of compound peak areas and the RSD between MMI_480_ and LIP. (C) Distribution of RSD ranges for lipids extracted by MMI_480_ and LIP. Peak area was normalized and log10-transformed; zero value was imputed with half of the minimum value before log10 transformation. Undefined RSD values caused by nondetected compounds (zero mean value) in one of the methods were replaced with 100 as a conservative imputation.

### Analysis of Certified Materials

To comprehensively evaluate the performance of MMI_480_, certified reference materials SRM 1950 and SRM 1957 were profiled and compared with established methods, yielding results consistent with previous findings. For exposomics/metabolomics, 175 compounds were identified in SRM 1950 and 132 compounds in SRM 1957 using both extraction methods. Compound intensities correlated well between the two approaches (*R* ^2^ = 0.772 for SRM 1950 and *R* ^2^ = 0.677 for SRM 1957) (Figure 3A). Eight amino acids listed in SRM 1950 (certified and noncertified) were detected, many of which were also identified in SRM 1957 using MMI_480_. The method further enabled the detection of multiple fatty acids reported in SRM 1950. Additionally, while bile acids were not included in the certified compounds, their detectability using MMI_480_ was comparable to ExMet and in agreement with previous reports (Tables S4 and S5).^22,23^ Regarding exogenous substances, six PFAS from SRM 1957 and seven from SRM 1950 were detected, which is aligned with previous studies.^24^ Other reported compounds, including steroids and disease-related metabolites, including creatinine and uric acid, were also identified using the new method. Notably, MMI_480_ yielded a higher number of compounds with RSD < 30% compared to ExMet in both reference materials (Figure 3B andFigures S7 and S8).

**Figure 3:**
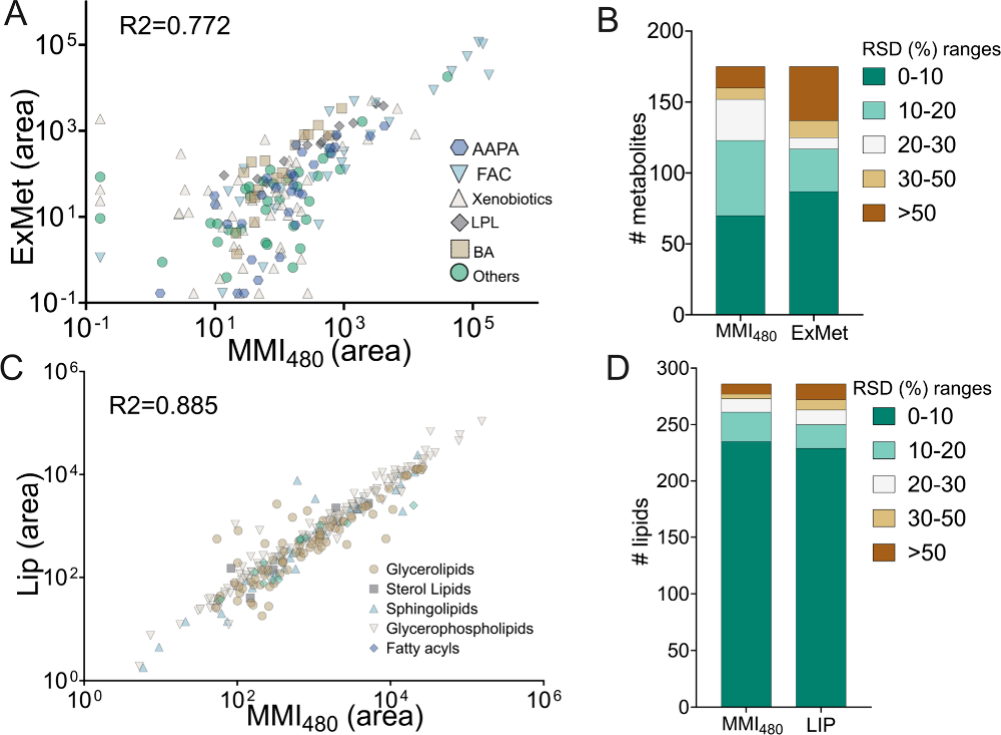
Metabolomics and lipidomics performance of MMI_480_ versus established methods (ExMet and LIP) in certified reference material SRM 1950. Scatter plots of metabolite and lipid peak areas compare MMI_480_ with ExMet (A) and LIP (C). Summary of RSD range distribution of compounds extracted from SRM 1950 using MMI480 and the established methods, ExMet (B) and LIP (D). Peak area was normalized and log10-transformed; zero value was imputed with half of minimum value before log10 transformation. Undefined RSD values caused by nondetected compounds (zero mean value) in one of the methods were replaced with 100 as a conservative imputation. AAPA: amino acid, peptides and analogues, FAC: fatty acids and conjugated, LPL: lysophospholipids, BA: bile acids.

For lipidomics, lipids extracted from SRM 1950 using MMI_480_ showed a strong correlation in peak areas with those obtained using the LIP extraction method (*R* ^2^ = 0.885). The method also achieved comparable performance with low RSDs across detected lipids relative to LIP (Figure 3C,D). Although certified reference materials do not provide detailed lipid composition, previous studies have reported lipid profiles of these materials. Most commonly detected lipid species from previous reports, including carnitine, TG, LPC, PC, LPE, PE, Cer, and SM, were also identified using the new method.^22,25,26^ All detected lipids and their classifications are presented in Table S6.

### Linearity and Quantification

We next assessed the linearity of three calibration curves developed for the quantification of BA and PFAS, polar metabolites, and lipids. Strong linearity was observed across all three calibration sets, with most coefficients of determination (*R* ^2^) consistently exceeding 0.99. PFAS showed excellent linearity in both the new and in-house methods (*R* ^2^ = 0.99), while BA demonstrated similar high linearity with comparable values between MMI_480_ (*R* ^2^ = 0.98–0.99) and ExMet (*R* ^2^ = 0.95–0.99). Amino acids also demonstrated good linearity (*R* ^2^ = 0.985–0.999 vs 0.978–0.99), except for aspartic acid, which had slightly lower values in both methods (0.93 for MMI480; 0.83 for ExMet), respectively. Most lipid standards, representing major lipid classes, displayed strong linearity in both MMI_480_ and LIP (*R* ^2^ = 0.987–0.999 vs 0.984–0.992, respectively). However, TG­(16:0/16:0/16:0) (*R* ^2^ = 0.81 vs 0.98) and TG­(18:0/18:0/18:0) (*R* ^2^ = 0.26 vs 0.94) exhibited poor linearity in MMI_480_. A detailed summary of the *R* ^2^ values for each standard is provided in Table S7.

Quantitative evaluation further showed that PFAS concentrations in SRM 1957 and SRM 1950 were generally in good agreement with certified values, except for PFNA in SRM 1950, and PFUnDA in both SRM 1950 and SRM 1957. For polar compounds, leucine, methionine, and phenylalanine exhibited concentrations closely matching the reported values, whereas threonine and valine showed notable deviations. These deviations were likely due to their low retention, which may have led to increased ion suppression and compromised quantitation. Although bile acids and lipids are not certified in SRM 1950, our quantification of bile acids was relatively consistent with previously reported data from 31 diverse laboratories, except for CA, TCDCA, and TDCA^25^ (Table 1 and Table S8). Additionally, lipid measurements showed partial agreement (e.g., LPC (18:0)) with the study by Mandal et al., which provided detailed acyl chain-level identification and quantitative data that closely matched the lipid standards analyzed in our work (Table 2 and Table S9).^22^

**Table 1:** Quantitative Values (ng/mL) of PFAS in SRM 1950 and SRM 1957 Using the New Method

	SRM 1950	reference values	SRM 1957	reference values
PFNA	0.50	0.705 (0.028)	0.17	0.878 (0.077)
PFOA	3.45	3.21 (0.6)	5.21	5.00 (0.44)
PFUnDA	0.44	0.182 (0.003)	0.29	0.172 (0.0036)
PFHxS	3.64	3.19 (0.08)	3.91	4.00 (0.83)
PFOS	9.20	10.43 (0.12)	21.19	21.1 (1.3)

**Table 2:** Quantitative Values of Selected Metabolites (ng/mL) and Lipids (μg/mL) in SRM 1950 Using the New Method

	SRM 1950	Reference/literature values	References
methionine	3.35	3.26 (0.26)	SRM 1950 certificate
phenylalanine	9.59	8.2 (1.1)	
proline	7.32	19.9 (1.1)	
threonine	2.33	13.94 (0.7)	
CA	0.22	0.12 (0.034)	Bowden et al.^25^
CDCA	0.33	0.3 (0.11)	
TDCA	0.07	0.04 (0.0064)	
UDCA	0.11	0.11 (0.024)	
LPE (18:1)	0.35	1.17 (0.16)	Mandal et al.^22^
LPC (18:0)	37.20	32.4 (16.5)	
SM (d18:1/16:0)	18.20	99.3 (31.5)	
PC (16:0–18:1)	107.93	139.29 (5.71)	
Cer (d18:1/18:1)	0.13	0.018 (0.017)	
PE (16:0/18:1)	2.04	1.33 (0.12)	
CE (16:0)	287.51	195 (106)	

### Cross-Laboratory Assessment

To evaluate cross-laboratory applicability, the MMI_480_ method was benchmarked against other established workflows by using different instrumentation. For metabolomics, compound intensities showed a remarkably high correlation between MMI_480_ and MeOH extraction (*R* ^2^ = 0.99; Figure 4A) with comparable RSDs. As expected, the MeOH method exhibited slightly better RSD performance, particularly for highly polar compounds, such as amino acids. However, MMI_480_ performed better in the extraction of both polar and semipolar lipids, such as LPC and LPE (Figure 4B). Additionally, MMI_480_ demonstrated a better extraction efficiency for environmental chemicals (Table S10). Compared with the modified Folch extraction method, MMI_480_ exhibited comparable lipid extraction performance, with good agreement in intensities (*R* ^2^ = 0.782) and RSDs across methods (Figure 4C,D). Notably, MMI_480_ performed slightly better than the Folch method in extracting (L)­PC, PE, and SM, further supporting its robustness as a versatile extraction method (Table S11).

**Figure 4:**
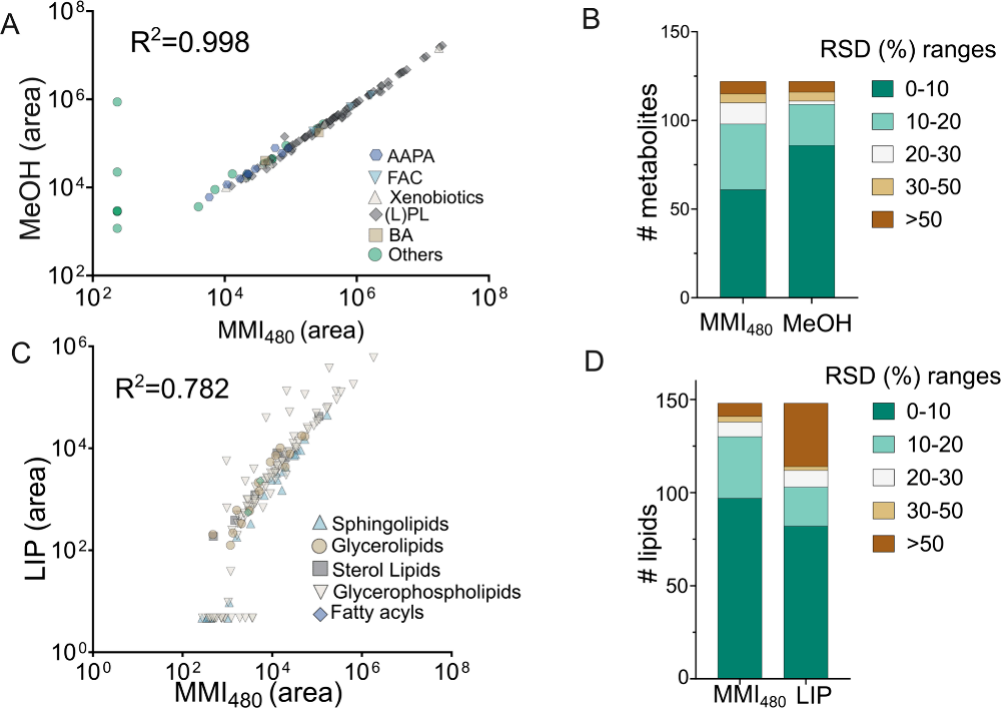
Cross-laboratory validation of MMI_480_ performance compared to established extraction methods. Metabolomics was benchmarked against MeOH extraction and lipidomics against the Folch method (CHCl_3_:MeOH, 2:1, v/v; LIP). (A) Scatter plots of metabolite peak areas and (B) RSD (%) comparing MMI_480_ with MeOH. (C) Scatter plots of lipid peak areas and (D) RSD comparing MMI_480_ with LIP. Peak area was normalized and log10-transformed; zero value was imputed with half of the minimum value before log transformation. AAPC: amino acid, peptides, and analogues, FAC: fatty acids and conjugated, (L)­PL: (Lyso)­phospholipids, BA: bile acids.

Although TG standards showed relatively unstable signals using the newly developed method across all tested samples, we observed that majority of the TGs were still well-detected, showing RSDs of less than 30% in all tested samples (*n* = 14–75), despite some individual compounds displaying higher RSD values. The relative contribution of each lipid class was also consistent with previous reports, indicating that glycerolipids (e.g., TG) and glycerophospholipids (e.g., PC and PE) are among the most abundant lipid classes.^26^ Additionally, we noticed that the TG species with lower reproducibility were eluted at the end of the 14 min gradient program (after 9 min of chromatography; Figure 5).

**Figure 5:**
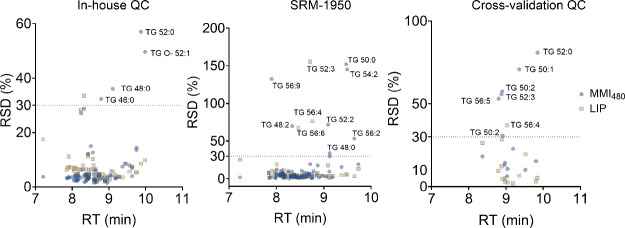
Relationship between retention time (RT) and relative standard deviation (RSD, %) of detected triglycerides (TG) across different extraction methods. Methods compared include the new MMI_480_, MeOH:MTBE:IPA (20:15:15, v/v/v), and Folch method (LIP). Data are shown for in-house quality control (QC) samples (*n* = 9), the reference material SRM 1950 (*n* = 3), and in-house QC samples from the cross-laboratory assessment (*n* = 3).

### Other Applications of the Proposed Method

Previous results have confirmed the suitability of MMI_480_ for the simultaneous extraction of analytes in exposomics, metabolomics, and lipidomics. We next aimed to evaluate whether MMI_480_ is also applicable to other analyte types including derivatized molecules (e.g., SCFAs and TCA cycle metabolites) and downstream proteomics.

### Analysis of Derivatized Compounds

To evaluate the compatibility of the method with derivatization, we extracted two reference materials, NIST Vegan and Omnivore, and derivatized the extracts using 3-NPH. Our results indicate that MMI_480_ enables the analysis of derivatized compounds, as evidenced by the detection of all four SCFA standards with relatively low variation (RSD: 2.2–11.5%). Additionally, approximately 40 annotated compounds exhibited comparable intensity and RSD values across both methods (Figure 6A), encompassing diverse chemical classes, including SCFAs, carbohydrates, organic acids, and amino acid derivatives. Notably, only a few studies have characterized the composition of NIST Vegan and Omnivore materials, particularly using derivatization to improve retention and signal intensity LC-ESI-MS.^27,28^ These characterization efforts represent valuable resources for ensuring verification of robustness across laboratories (Tables S12 and S13).

**Figure 6:**
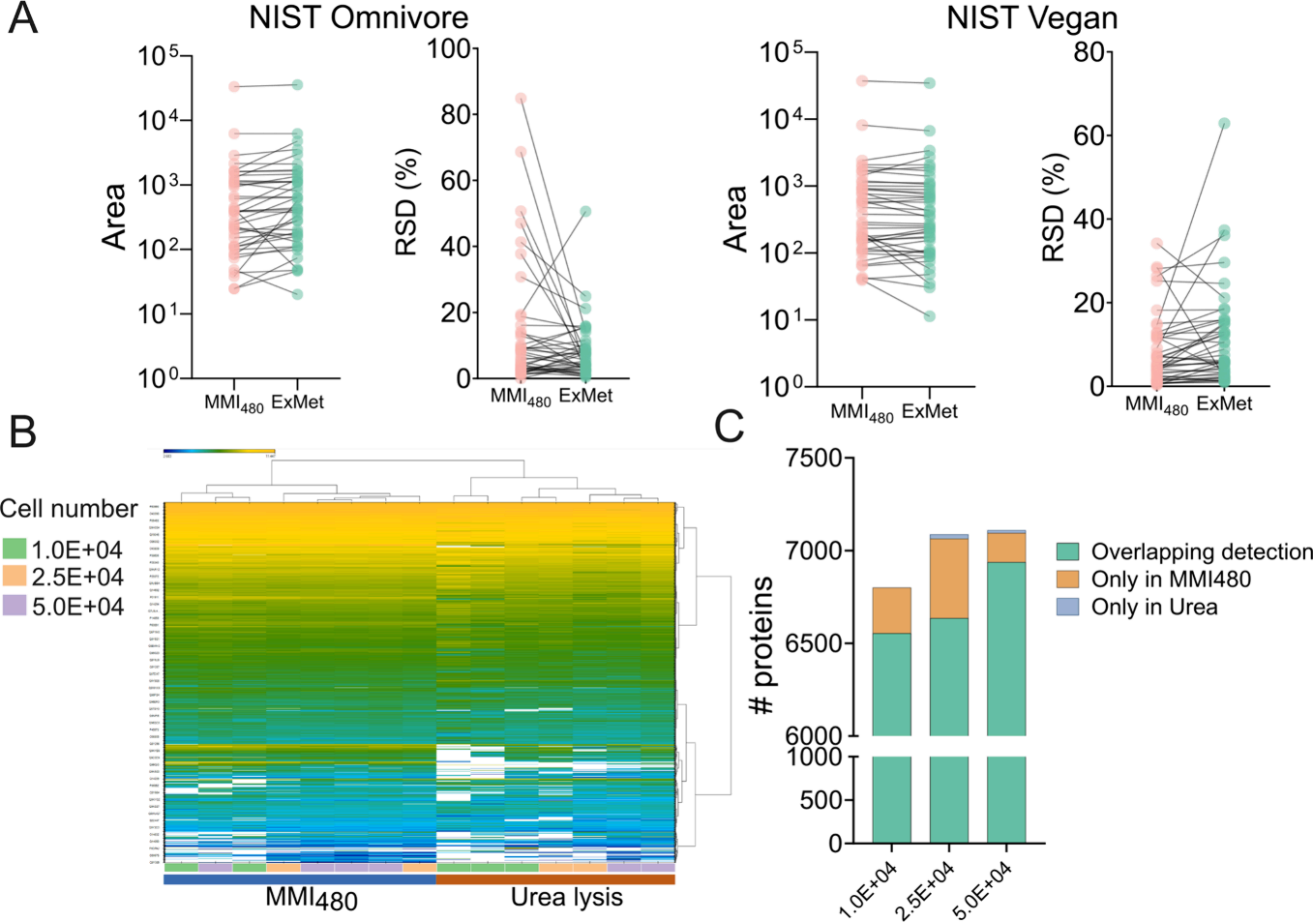
Versatile applications of the novel MMI_480_ method for extracting derivatized compounds for LC-MS and proteomics analysis. For derivatization, two reference materials, NIST Omnivore and NIST Vegan, were compared using MMI_480_ and the exposomics/metabolomics (ExMet) method. (A) Comparison of peak areas and relative standard deviation (RSD, %) of derivatized compounds detected in the two stool samples with MMI_480_ and ExMet. (B) Proteomics results from A375 cell lines extracted with MMI_480_ compared with the urea lysis method. (C) Number of detected proteins in each method. Peak area was normalized and log10-transformed.

### Proteomics Analysis

For proteomics, we assessed the suitability of the MMI_480_ method using the A375 cell line. To examine its performance across different sample amounts, we extracted and compared three cell counts (1.0 × 10^05^, 2.5 × 10^05^, and 5.0 × 10^05^) between MMI_480_ and a standard urea lysis method. Our findings indicate that MMI_480_ exhibits performance comparable to the urea lysis method with regard to number of protein annotations, particularly at lower cell numbers (Figure 6B,C). Notably, some proteins appeared to be better extracted with the MMI_480_ method, suggesting that the MMI extraction buffer can solubilize certain proteins more effectively (Table S14).

### Limitation of the Proposed Method

As mentioned above, the proposed method offers many advantages and is capable of capturing compounds with a broad spectrum of chemical properties. However, its application was explored primarily for use in blood-derived samples, stool, and cell culture, leaving its performance for tissue samples yet to be evaluated. Notably, a subset of late-eluting glycerolipids in the applied LC-HRMS method appeared less reproducible using this method, indicating the need for further adjustment to consider these selected compounds. The current work assessed the method using a single LC gradient and polarity for each of the exposomics/metabolomics and lipidomics workflows. For more comprehensive chemical coverage, dual ionization modes for both analytical methods can be applied. Our in-house LC-MS method was intentionally optimized for bile acids (BAs), whose amphipathic and surface-active properties are known to exhibit distinct ionization behavior compared with other metabolite classes, even when retention times overlap. Although additional isotope-labeled ISTDs were included (e.g., amino acids, a fatty acid, and several PFAS), the overall ISTD panel remained biased toward BAs to maximize quantitative robustness for that class. We acknowledge that this class-focused ISTD coverage may not provide equally optimal normalization across all chemical classes, which represents a limitation when comparing the in-house method to a broadly targeted platform. Finally, future studies evaluating its performance using other separation techniques (e.g., HILIC and SFC) would also provide more information about the chemical coverage of the method. In this method development setting, we analyzed pooled human plasma quality control samples and standard reference materials and included solvent blanks, extraction blanks, and standard compound mixtures; however, pooled quality control samples were not analyzed, as these are primarily essential for monitoring analytical drift and data quality in large-scale biological cohort studies. Overall, the novel method offers a simple, efficient, and scalable approach for simultaneous metabolomics and lipidomics analyses. For liquid samples, the method is entirely compatible with the well-plate format adaptation. Due to its monophasic nature, the method is easily transferable to (semi)­automated platforms for biofluid extraction, making it particularly well-suited for high-throughput workflows, especially in large-scale clinical studies.

## Conclusions

This study introduces a novel single-extraction workflow, named SIMPLIFY, that achieves performance comparable to conventional (and isolated) approaches for metabolomics/exposomics, lipidomics, and proteomics, thus offering a simplified and eco-friendly procedure. Its simplicity, flexibility, and ability to enhance analytical efficiency and streamline extraction processes make this method particularly suitable for large-scale studies and multiomics infrastructure platforms.

## Supplementary Material





## Notes

Experimental details, materials, and methods; performance of MeOH:MTBE:IPA (20:15:15, v/v/v) in exposomics/metabolomics across specific lipid classes from four different ratios, compared to the ACN protein precipitation method (ExMet); performance of MeOH:MTBE:IPA (20:15:15, v/v/v) in lipidomics across specific lipid classes from four different ratios, compared to the Folch extraction method (LIPI); and relationship between retention time and relative standard deviation (RSD) of detected triglycerides (TG) in the new method, MeOH:MTBE:IPA (20:15:15,v/v/v), and the Folch method (LIP) across three extractions: QC sample (QC), reference material (SRM-1950), and QC in cross-validation assessment (Crossvalidation) (PDF)
Main Mzmine parameters for data processing; amino acids, peptides, and analogues; list of detected lipids across four extraction ratios compared with the established lipidomics method; and list of detected compounds in SRM 1950 extracted using the MMI480 method and the established exposomics/metabolomics (ExMet) method (XLSX)
