# Supplementary material for: The SIMPLIFY Protocol: A Monophasic Extraction System Suitable for Exposomics, Metabolomics, Lipidomics, and Proteomics Research
Source: Anal Chem. 2025 Nov 19;97(47):26175–83. doi: 10.1021/acs.analchem.5c05322 (PMC12676512; doi:10.1021/acs.analchem.5c05322)
Supplement: Supplementary file 1 [file ac5c05322_si_001.pdf]

Supplementary file

The SIMPLIFY Protocol: A Monophasic Extraction System Suitable for Exposomics, Metabolomics, Lipidomics, and Proteomics Research

Anh Hoang Nguyen[a], Victor Castro-Alves*[a], Emilia Holmström Flores[a], João Marcos G. Barbosa[a], Matilda Kråkström[b], Päivikki Reinivuori[b], Otto Kauko[b], Alex Dickens[b,c], Matej Orešič[b,d,e], Tuulia Hyötyläinen[a,*]

[a]*Man-Technology-Environment (MTM) Research Centre, School of Science and Technology, Örebro University, SE-701 82 Örebro, Sweden*

[b]*Turku Centre for Biotechnology, University of Turku and Åbo Akademi University, FI-20520 Turku, Finland*

[c]*Department of Chemistry, University of Turku, Finland, FI-20500 Turku, Finland*

[d]*Department of Life Technologies, University of Turku, FI-20014 Turku, Finlan*

[e]*School of Medical Sciences, Faculty of Medicine and Health, Örebro University, SE-701 82 Örebro, Sweden*

TABLE OF CONTENT:

SUPPLEMENTARY METHODS

- **Cross-Laboratory Validation**
- **Metabolomics Analysis.**
- **Lipidomics Analysis.**
- **Proteomics Preparation and Analysis**
- **Sample Preparation with Urea Lysis.**
- **LC-ESI-MS/MS Analysis.**
- **Data Analysis.**

SUPPLEMENTARY FIGURES

- **Figure S1.** Performance of MeOH:MTBE:IPA (20:15:15, v/v/v) in exposomics/metabolomics across specific lipid classes from four different ratios , compared to the ACN protein precipitation method (ExMet).
- **FigureS2.** Performance of MeOH:MTBE:IPA (20:15:15, v/v/v) in lipidomics across specific lipid classes from four different ratios, compared to the Folch extraction method (LIPI).
  Figure S3. The relationship between retention time and relative standard deviation (RSD) of detected triglycerides (TG) in the new method, MeOH:MTBE:IPA (20:15:15, v/v/v), and the Folch method (LIP) across three extractions: QC sample (QC), reference material (SRM-1950), and QC in cross-validation assessment (Cross-validation).

SUPPLEMENTARY TABLES

- **Table S1.** Main Mzmine parameters for data processing
- **Table S2. Linearity of calibration mixtures**
- **Table S3.** Quantitative values (ng/mL) of PFAS in SRM-1950 and SRM-1957 using the new method
- **Table S4.** Quantitative values of metabolites (ng/mL) and lipids (µg/mL) in SRM-1950 using the new method, in comparison with either reference values (for amino acids) or balues reported in other studies.

SUPPLEMENTARY METHODS

Analysis of Derivatization, SCFA, and TCA

Samples were analyzed using an ultra-high-performance liquid chromatography (UHPLC) system (Agilent Technologies, Santa Clara, USA) coupled to a quadrupole time-of-flight mass spectrometer (qToF-MS, 6545 series). A sample volume of 5 µL was injected onto an Acquity UPLC BEH C18 column (2.1 × 100 mm, 1.7 µm; Waters, Milford, USA). The mobile phase consisted of 0.1% (v/v) formic acid in water (solvent A) and acetonitrile (solvent B). The elution gradient was set at a flow rate of 0.4 mL/min as follows: 10% B from 0 to 2 minutes, a linear increase to 100% B from 2 to 4 minutes, 100% B from 4 to 6 minutes, followed by re-equilibration at 10% B for 4 minutes. The column temperature was maintained at 50 °C, and the autosampler was kept at 10 °C.

Mass spectrometry was performed in negative ion mode with the following parameters: a scan rate of two spectra per second and an m/z range of 100–1000. The dual electrospray ionization (ESI) source parameters were set as follows: 0 V collision energy, 3.6 kV capillary voltage, 1500 V nozzle voltage, a nebulizer pressure of 21 psi, a sheath gas flow rate of 10 L/min, and a sheath gas temperature of 379 °C. Data acquisition and conversion to mzData format for preprocessing were conducted using MassHunter Workstation Software.

Cross-Laboratory Validation

Metabolomics Analysis. Samples were extracted using ice-cold methanol or a methanol:methyl tert-butyl ether:isopropanol (MeOH:MTBE:IPA) mixture, with internal standards including glutamic acid-d5, stearic acid-d35, succinic acid-d4, and valine-d8. A 10 µL aliquot of the extracted sample was injected onto an Acquity BEH C18 column (2.1 × 100 mm, 1.7 µm). The LC gradient was applied at a flow rate of 0.4 mL/min as follows: 100% A from 0 to 2 minutes, 0% A from 2 to 10 minutes, 100% A from 10 to 10.1 minutes, followed by equilibration at 100% A until 16 minutes. The column temperature was maintained at 50 °C, and the sample temperature at 10 °C.

Data acquisition was performed in data-dependent analysis mode using the ZenoTOF 7600 system. The source temperature was set to 500 °C, with a mass range of 50–1000 for both MS1 and MS2. MS1 accumulation time was set to 100 ms, with a curtain gas pressure of 45 psi and a spray voltage of -4500 V.

Lipidomics Analysis. Lipid extraction was performed using the Folch method with a chloroform:methanol (2:1, v/v) mixture or a methanol:MTBE:IPA mixture containing the following internal standards: C15 ceramide-d7, C13-dihydroceramide-d7, 16:0 cholesteryl-d7 ester, 1-pentadecanoyl-2-oleoyl(d7)-sn-glycero-3-phosphocholine (15:0-18:1-d7-PC), 1-pentadecanoyl-2-oleoyl(d7)-sn-glycero-3-phosphoethanolamine (15:0-18:1-d7-PE), 1-oleoyl(d7)-2-hydroxy-sn-glycero-3-phosphocholine (18:1-d7 Lyso PC), 1-oleoyl(d7)-2-hydroxy-sn-glycero-3-phosphoethanolamine (18:1-d7 Lyso PE), 1-pentadecanoyl-2-oleoyl(d7)-sn-glycerol (15:0-18:1-d7 DG), 1,3-dipentadecanoyl-2-oleoyl(d7)-glycerol (15:0-18:1-d7-15:0 TG), and N-oleoyl-D-erythro-sphingosylphosphorylcholine-d9 (18:1-d9 SM).

A 1 µL volume of the extracted lipid sample was injected into the UPLC® BEH C18 column (2.1 mm × 100 mm, particle size 1.7 µm) by (Waters, Milford, USA). The mobile phase includes Water + 1% NH4Ac (1M) + 0.1% HCOOH (A) and ACN:IPA (1:1, v/v) + 1% NH4Ac + 0.1% HCOOH (B). The LC gradient was applied at a flow rate of 0.4 mL/min as follows: 0 to 2 min 35-80% B, 2 to 7 min 80-100% B and 7 to 14 min 100% B. The column temperature was set at 50 °C, and the sample temperature at 10 °C. Full-scan mode was used with the TripleTOF 6600 DuoSpray Ion Source (AB Sciex Instruments), while data was acquired in data-dependent analysis mode with the ZenoTOF 7600 system. The source temperature was

set at 650 °C, with a mass range of 100–1700 for MS1 and an MS1 accumulation time of 250 ms.

Proteomics Preparation and Analysis

Sample Preparation with Urea Lysis. Samples were lysed in 8M urea with 50 mM Tris at pH 8. Reduction was performed using dithiothreitol (DTT) at a final concentration of 10 mM, followed by alkylation with iodoacetamide (IAA) at a final concentration of 20 mM. The buffer was then diluted to achieve a urea concentration of <2M before enzymatic digestion. Digestion was carried out overnight at room temperature using trypsin (Promega). After digestion, peptides were desalted using Sep-Pak tC18 cartridges (Waters), evaporated to dryness, and stored at -20 °C until analysis.

LC-ESI-MS/MS Analysis. Liquid chromatography-electrospray ionization tandem mass spectrometry (LC-ESI-MS/MS) analysis was performed using an Evosep One HPLC system (Evosep, Odense, Denmark) coupled to a timsTOF fleX mass spectrometer (Bruker, Bremen, Germany) equipped with a CaptiveSpray nano-electrospray ionization source. Peptide separation was conducted on an 8 cm Evosep Performance column (150 µm × 8 cm, 1.5 µm C18-beads, EV1109) using a mobile phase composed of water with 0.1% formic acid (solvent A) and 0.1% formic acid/99.9% acetonitrile (v/v) (solvent B). The 21-minute gradient method was used, allowing the analysis of 60 samples per day.

Peptides were analyzed using a data-independent acquisition (DIA) LC-MS/MS method, with automated MS data acquisition performed using Compass 2025 software (Bruker). The mass spectrometer was operated in dia-PASEF mode, utilizing the default “short gradient method.”

Data Analysis. Raw data were analyzed using Spectronaut software (Biognosys; version 19.4.241104.62635). Protein identification was performed using the DirectDIA approach, and label-free quantification was conducted using MaxLFQ. The main data analysis parameters in Spectronaut included the use of trypsin/P as the digestion enzyme, allowing up to two missed cleavages. Fixed modifications included carbamidomethylation, while variable modifications included N-terminal acetylation and methionine oxidation. Protein identification was performed against the Swiss-Prot 2024_04 Homo sapiens database and the Universal Protein Contaminant database[1]. The precursor and protein false discovery rate (FDR) cutoffs were set at 0.01. Quantification was based on the area under the curve within integration boundaries for each targeted ion, and normalization was performed using local normalization based on a retention-time-dependent local regression model, as described by Callister et al.[2] (2006).

SUPPLEMENTARY FIGURES

Figure S1. Performance of MeOH:MTBE:IPA (20:15:15, v/v/v) in exposomics/metabolomics across specific lipid classes from four different ratios , compared to the ACN protein precipitation method (ExMet).

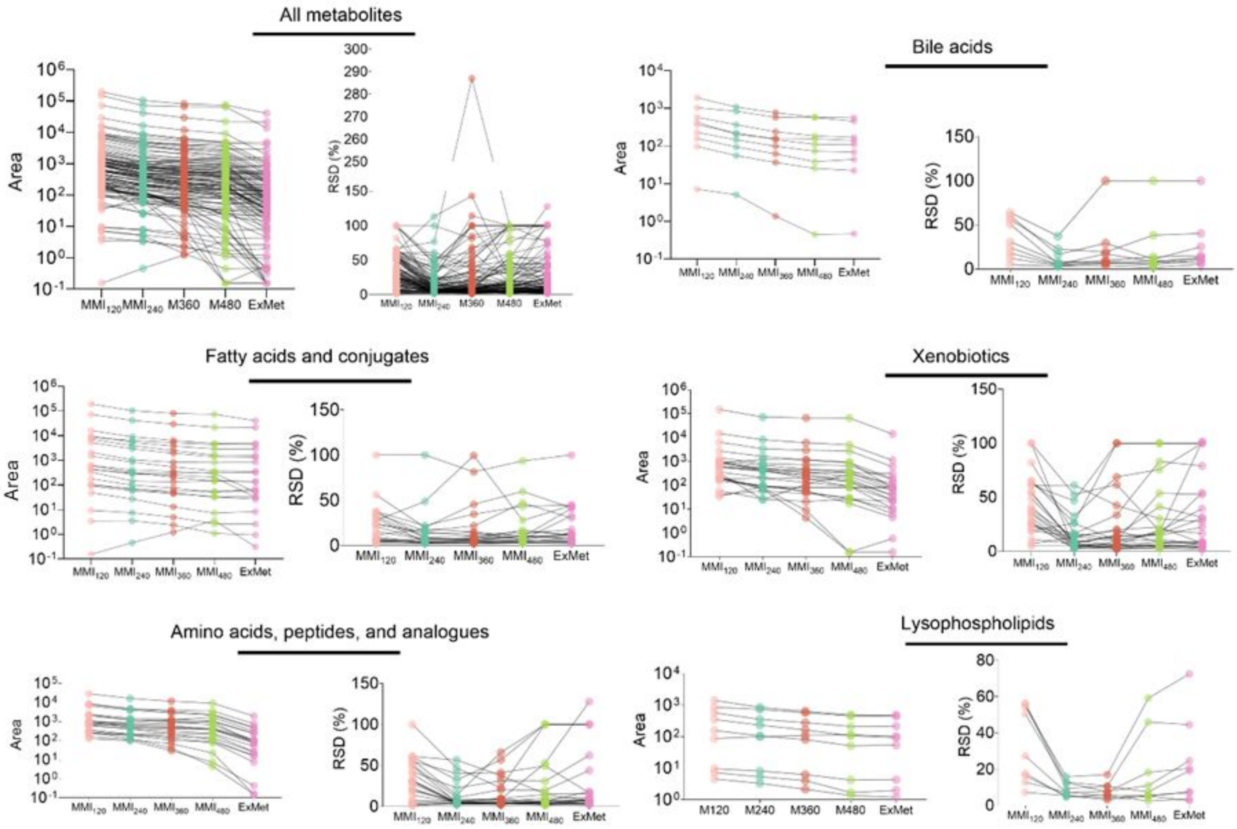
All metabolites
Bile acids
Fatty acids and conjugates
Xenobiotics
Amino acids, peptides, and analogues
Lysophospholipids
Area
RSD (%)
MMI120
MMI240
M360
M480
MMI360
MMI480
M120
M240
ExMet

FigureS2. Performance of MeOH:MTBE:IPA (20:15:15, v/v/v) in lipidomics across specific lipid classes from four different ratios, compared to the Folch extraction method (LIPI).

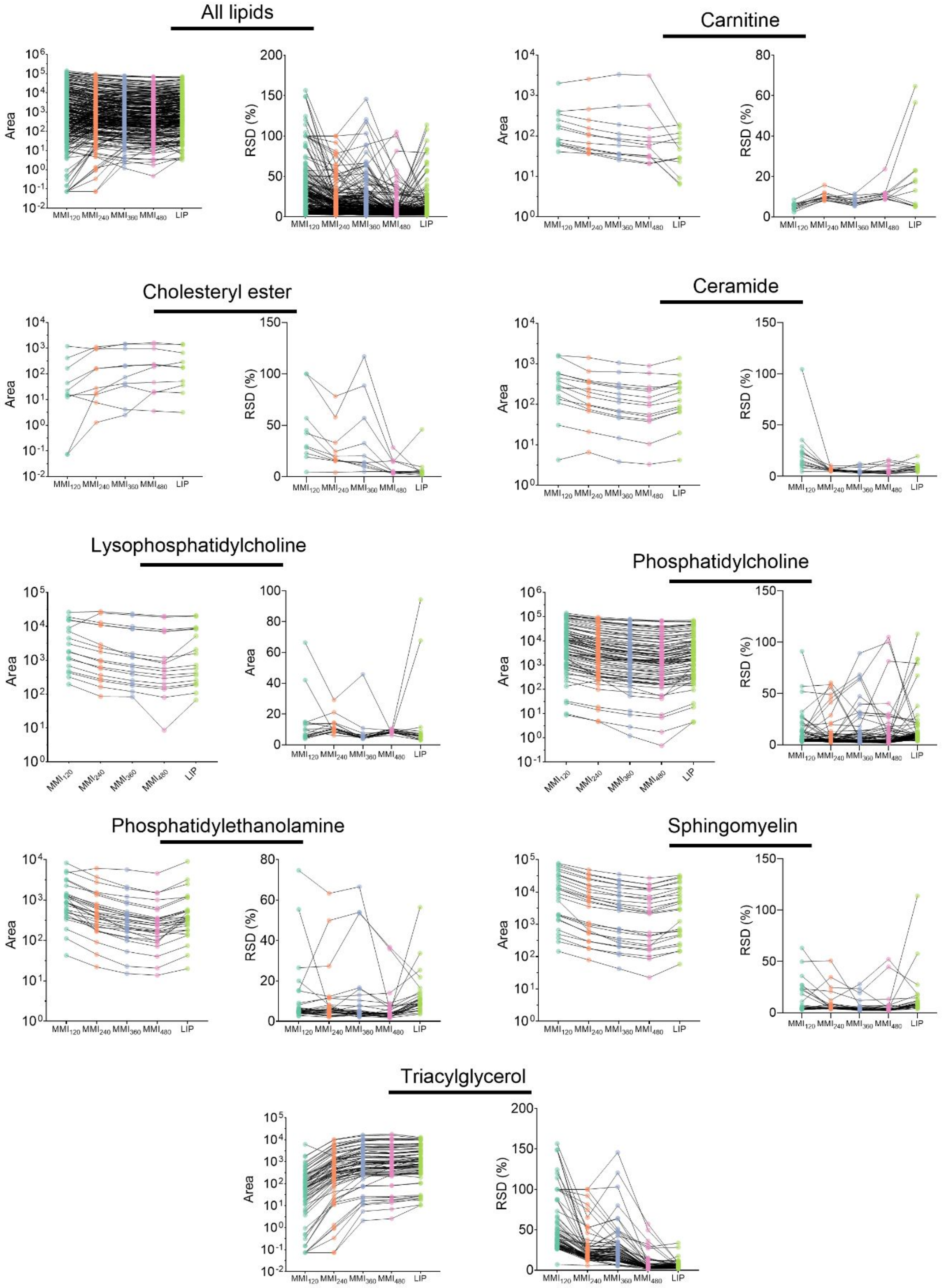

Figure S3. The relationship between retention time and relative standard deviation (RSD) of detected triglycerides (TG) in the new method, MeOH:MTBE:IPA (20:15:15, v/v/v), and the Folch method (LIP) across three extractions: QC sample (QC), reference material (SRM-1950), and QC in cross-validation assessment (Cross-validation).

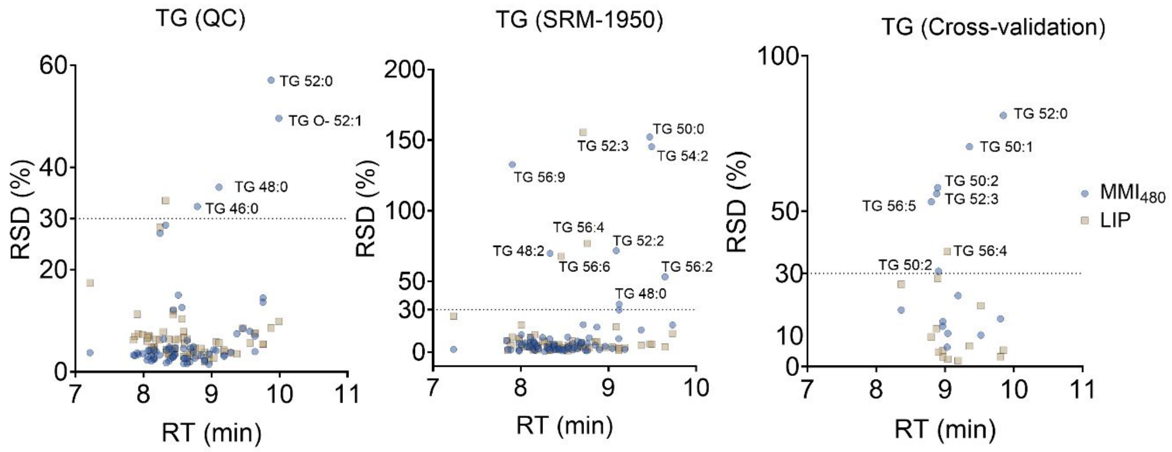

SUPPLEMENTARY TABLES

Table S1. Main Mzmine parameters for data processing

**Parameters**	
Mass detection	MS1 (1.5E3)
**Chromatogram builder**	
Scan filter	MS1
Minimum consecutive scan	5
Minimum intensity	1.0E3
Minimum absolute height	1.5E3
m/z tolerance	0.007 (7 ppm)
**Local minimum resolver**	
Chromatographic threshold	70%
Minimum RT search range	0.08
Absolute height	1.5E3
Peak top/edge	1.05
RT duration	0.01-1.2
**Alignment**	
m/z tolerance	0.009 (8 ppm)
Weight for m/z	3
RT tolerance	0.15
Weight for RT	1
**Peak finder**	
Intensity tolerance	10%
m/z tolerance	0.01 (10 ppm)
RT tolerance	0.1
**Identification**	
m/z	0.009 (10 ppm)
RT	0.2

Table S2. Linearity of calibration mixtures

Standards	MeOH:MTBE:IPA (MMI_{480})	Metabolomics (ACN) and Lipidomics (Folch)
**Lipids**		
CE (18:1)	0.987	0.993
Cer (d18:1/18:1)	0.990	0.990
LPC (18:0)	0.995	0.993
LPC (18:0)	0.995	0.988
LPC (18:1)	0.999	0.993
LPC (18:1)	0.997	0.990
LPE (18:1)	0.997	0.984
PC (16:0-18:1)	0.993	0.990
PC (16:0/16:0)	0.993	0.990
PC (18:0/18:0)	0.991	0.987
PE (16:0/18:1)	0.992	0.982
TG (16:0/16:0/16:0)	0.811	0.982
TG (18:0/18:0/18:0)	0.264	0.942
**Bile acids**		
THCA	0.993	0.998
GHDCA	0.997	0.997
HCA	0.998	0.997
HDCA	0.990	0.989
GCDCA	0.997	0.996
GDCA	0.998	0.999
w/a-MCA	0.999	0.999
TaMCA	0.999	1.000
GUDCA	0.998	0.998
THDCA	0.980	0.990
GLCA	1.000	0.999
GCA	0.991	0.998
UDCA	0.995	0.997
CA	0.999	0.998
7-oxo-DCA	0.999	1.000
TLCA	1.000	0.999
GHCA	0.999	0.998
LCA	0.986	0.992
TCA	0.998	0.998
DCA	0.998	0.956
**Perfluoroalkyl and Polyfluoroalkyl Substances**		
PFHxS Linear	0.999	0.999
PFOA	0.999	0.998
PFUnDA	0.999	0.999
PFNA	0.996	0.987
PFOS Branched	0.999	0.999
**Polar metabolites**		
Abscisic acid	0.993	0.999
Caffeic acid	0.991	0.991
Hydroxyglutaric acid	0.968	0.959
Naringenin	0.958	0.983
Phenylalanine	1.000	0.998
Salicylic acid	0.998	0.988
Succinic acid	0.992	0.985
Catechin	0.968	0.959
Sorbitol	0.986	0.984

Sucrose	0.990	0.996
Vanillic acid	0.999	0.992
Ferulic acid	0.998	0.996
3-Carboxy-4-methyl-5-propyl-2-furanpropanoic acid (CMPF)	0.997	0.999
5-Oxoproline	0.999	0.999
Coumaric acid	0.996	0.979
Gibberelic acid	0.996	0.997
Glutamic acid	0.996	0.996
Glutamine	0.997	0.998
Hippuric acid	0.998	0.996
Indole-3-propionic acid	0.993	0.998
Inosine	0.999	0.998
Kinetin	0.997	0.984
Leucin	0.997	0.997
Mevalonic acid	0.995	0.998
Proline	0.999	0.998
Sinapic acid	0.998	0.998
Taurine	0.997	0.998
tryptophan	0.998	0.996
Valine	0.999	0.999
2-Hydroxybutyric acid	0.988	0.997
4-Hydroxybenzyl alcohol	0.997	0.993
Asparagine	0.993	0.978
Dihydroxy caffeic acid	0.977	0.988
Kynurenic acid	0.998	0.997
p-cresol	0.984	0.975
Threonine	0.990	0.992
Lysine	0.999	0.351
Dihydroxy caffeic acid	0.993	0.953
Indole-3-acetic acid	0.993	0.999
Lactic acid	0.990	0.989
Methionine	0.987	0.997
Shikimic acid	0.919	0.992
Rosmarinic acid	0.935	0.982
Galacturonic acid	0.963	0.749
Aspartic acid	0.933	0.865
Gallic acid	0.892	0.857

Table S3. Quantitative values (ng/mL) of PFAS in SRM-1950 and SRM-1957 using the new method

	**SRM-1950**	**Reference values**	**SRM-1957**	**Reference values**
PFNA	0.50	0.705 (0.028)	0.17	0.878 (0.077)
PFOA	3.45	3.21 (0.6)	5.21	5.00 (0.44)
PFUnDA	0.44	0.182 (0.003)	0.29	0.172 (0.0036)
PFHxS	3.64	3.19 (0.08)	3.91	4.00 (0.83)
PFOS	9.20	10.43 (0.12)	21.19	21.1 (1.3)

Table S4. Quantitative values of metabolites (ng/mL) and lipids (µg/mL) in SRM-1950 using the new method, in comparison with either reference values (for amino acids) or balues reported in other studies.

	SRM-1950	Reference* or reported # values	References
Leucine	10.06	12.90 (0.82)*	SRM-1950
Methionine	3.35	3.26 (0.26) *	
Phenylalanine	9.59	8.2 (1.1) *	
Proline	7.32	19.9 (1.1) *	
Threonine	2.33	13.94 (0.7) *	
Valine	13.69	20.9 (1.2) *	
CA	0.22	0.12 (0.034) #	Bowden et al.[3]
CDCA	0.33	0.3 (0.11) #	
DCA	0.37	0.35 (0.083) #	
GCA	0.31	0.24 (0.069) #	
GDCA	0.39	0.43 (0.18) #	
GCDCA	1.03	1.1 (0.069) #	
GLCA	0.03	0.025 (0.0018) #	
GUDCA	0.15	0.15 (0.024) #	
LCA	0.01	0.014 (0.0036) #	
TCA	0.02	0.026 (0.0056) #	
TCDCA	0.16	0.084 (0.005) #	
TDCA	0.07	0.04 (0.0064) #	
UDCA	0.11	0.11 (0.024) #	
LPC (18:1)	35.27	23.8 (10.2) #	Mandal et al.[4]
LPE (18:1)	0.35	1.17 (0.16) #	
LPC (18:0)	37.20	32.4 (16.5) #	
SM (d18:1/16:0)	18.20	99.3 (31.5) #	
PC (16:0-18:1)	107.93	139.29 (5.71) #	
PC (16:0/16:0)	13.25	9.88 (0.46) #	
Cer (d18:1/18:1)	0.13	0.018 (0.017) #	
PE (16:0/18:1)	2.04	1.33 (0.12) #	
PC (18:0/18:0)	0.57	1 (0.2) #	
CE (16:0)	287.51	195 (106) #	

References

1 . Frankenfield, A. M.; Ni, J.; Ahmed, M.; Hao, L. *J Proteome Res* **2022**, *21* (9), 2104-2113.
2 . Callister, S. J.; Barry, R. C.; Adkins, J. N.; Johnson, E. T.; Qian, W. J.; Webb-Robertson, B. J.; et al. *J Proteome Res* **2006**, *5* (2), 277-286.
3 . Bowden, J. A.; Heckert, A.; Ulmer, C. Z.; Jones, C. M.; Koelmel, J. P.; Abdullah, L.; et al. *J Lipid Res* **2017**, *58* (12), 2275-2288.
4 . Mandal, R.; Zheng, J.; Zhang, L.; Oler, E.; LeVatte, M. A.; Berjanskii, M.; et al. *Anal Chem* **2025**, *97* (1), 667-675.
